# Effectiveness of a computerized clinical decision support system for prevention of glucocorticoid-induced osteoporosis

**DOI:** 10.1038/s41598-022-19079-7

**Published:** 2022-09-02

**Authors:** Toru Morikawa, Mio Sakuma, Tsukasa Nakamura, Tomohiro Sonoyama, Chisa Matsumoto, Jiro Takeuchi, Yoshinori Ohta, Shinji Kosaka, Takeshi Morimoto

**Affiliations:** 1 Department of Clinical Epidemiology, Hyogo Medical University, 1-1 Mukogawa, Nishinomiya, Hyogo 663-8501 Japan; 2 Department of General Medicine, Nara City Hospital, Nara, Japan; 3 Department of Infectious Diseases, Shimane Prefectural Central Hospital, Izumo, Japan; 4 Department of Pharmacy, Shimane Prefectural Central Hospital, Izumo, Japan; 5 Center for Health Surveillance and Preventive Medicine, Tokyo Medical University, Tokyo, Japan; 6 Shimane Prefectural Central Hospital, Izumo, Japan

**Keywords:** Health care, Risk factors

## Abstract

Glucocorticoids are widely used for a variety of diseases, but the prevention of glucocorticoid-induced osteoporosis is sometimes neglected. Therefore, the effectiveness of a computerized clinical decision support system (CDSS) to improve the performance rate of preventive care for glucocorticoid-induced osteoporosis was evaluated. We conducted a prospective cohort study of outpatients who used glucocorticoids for three months or longer and who met the indication for preventive care based on a guideline. The CDSS recommended bisphosphonate (BP) prescription and bone mineral density (BMD) testing based on the risk of osteoporosis. The observation period was one year (phase 1: October 2017–September 2018) before implementation and the following one year (phase 2: October 2018–September 2019) after implementation of the CDSS. Potential alerts were collected without displaying them during phase 1, and the alerts were displayed during phase 2. We measured BP prescriptions and BMD testing for long-term prescription of glucocorticoids. A total of 938 patients (phase 1, 457 patients; phase 2, 481 patients) were included, and the baseline characteristics were similar between the phases. The median age was 71 years, and men accounted for 51%. The primary disease for prescription of glucocorticoids was rheumatic disease (28%), followed by hematologic diseases (18%). The prevalence of patients who needed an alert for BP prescription (67% vs. 63%, P = 0.24) and the acceptance rate of BP prescription (16% vs. 19%, P = 0.33) were similar between the phases. The number of patients who had orders for BMD testing was significantly increased (4% vs. 24%, P < 0.001) after CDSS implementation. The number of patients who needed an alert for BMD testing was significantly decreased from 93% in phase 1 to 87% in phase 2 (P = 0.004). In conclusion, the CDSS significantly increased BMD testing in patients with a higher risk of glucocorticoid-induced osteoporosis, but did not increase BP prescription.

## Introduction

Clinical practice guidelines (CPG) have been considered to have a central role in improving the quality and efficiency of healthcare^1^. However, the implementation rate of CPGs has not been high^2^ and it has been challenging to provide guideline-based medical care in the primary care field because of the wide range of areas covered^3^. Compared to specialists who are more likely to implement CPGs because of the focused range of their field, it was challenging for generalists to implement a CPG for patients with multiple comorbid diseases^4^.

Computerized clinical decision support systems (CDSSs) have been reported to provide evidence-based recommendations based on patient-specific information and enhance clinical performance for drug dosing and preventive care^5^ CDSSs have been reported to increase prophylaxis of venous thromboembolism in inpatients^6,7^ and improve documentation of asthma care in the emergency room^8^. CDSSs provide assessments or recommendations similar to those from expert physicians based on the CPG^9^. These changes in practice with CDSS were reported for predetermined preventive care or documentation of risk populations, but the change in management based on the individual treatment status according to a CPG using a CDSS has not been reported.

Glucocorticoids are widely used for a variety of diseases, but the prevention of glucocorticoid-induced osteoporosis has been challenging. Although the treatment of glucocorticoid-induced osteoporosis is well established, and a CPG for this is widely recognized, management of glucocorticoid-induced osteoporosis is suboptimal^10,11^. CDSSs have been widely utilized for the management of primary osteoporosis^12^, but not for the management of glucocorticoid-induced osteoporosis. The management of glucocorticoid-induced osteoporosis is more complicated because physicians of many subspecialties are involved, and the CPG is not straightforward. Several interventions other than CDSSs, such as direct feedback or education, were reported, but the effectiveness of CDSSs in the management of glucocorticoid-induced osteoporosis was not reported^13,14^.

We thus hypothesized that CDSSs could improve adherence to the CPG for the prevention of glucocorticoid-induced osteoporosis, developed a CDSS for glucocorticoid-induced osteoporosis, and evaluated its effectiveness for improving management based on the rates of BP prescription and BMD testing in a prospective cohort study.

## Methods

### Study design and patient population

We conducted a prospective cohort study at a tertiary care teaching hospital in Japan. It had 618 beds with 28 departments equipped with electronic medical records and a computerized ordering system. The study was conducted from October 1, 2017 to September 30, 2019, and the computerized clinical decision support system (CDSS) with guideline-based glucocorticoid-induced osteoporosis management was implemented on October 1, 2018. The study period was divided into 1 year before implementation (phase 1: October 2017–September 2018) and 1 year after implementation (phase 2: October 2018–September 2019).

We retrieved clinical data for all outpatients, and those who met criteria for bisphosphonate (BP) prescription at any point during the study period were included. The criteria for BP prescription were derived from the guideline for glucocorticoid-induced osteoporosis issued by the Japanese Society for Bone and Mineral Research^15^. It advised that patients who took a glucocorticoid for more than 3 months should be prescribed a BP based on their risks, and we modified the guideline to develop the inclusion criteria and the CDSS to be easily applicable in daily clinical practice (Supplementary Table S1). The key factors for recommendation were duration and dose of glucocorticoid, age, and bone mineral density (BMD). The dose of glucocorticoid was converted to the mg equivalent of prednisolone (PSL). All BP were oral formulations and intravenous BP formulations were not included in this study.

The CDSS also recommended BMD testing if it had not been done in the past year. The skeletal areas for BMD testing were either spine, proximal femur, or both according to the physician in charge.

The index date was the day of glucocorticoid prescription when the duration of glucocorticoid prescription became 3 months or longer. Patients had multiple index dates if they were prescribed glucocorticoids at outpatient clinics more than once during the study period when the glucocorticoids were calculated to be administered for 3 months or longer at each prescription. When the index date was before October 1st, 2018, such patients were included in phase 1, and patients whose index date was October 1st, 2018 or later were included in phase 2. Although some patients were followed beyond October 1st, 2018 and each index day of glucocorticoid prescription was included in phase 1 or phase 2, each patient was included in either phase 1 or phase 2 according to the earliest index day.

This study was approved by the institutional review boards at Hyogo College of Medicine and Shimane Prefectural Central Hospital. According to the policy of the Shimane Prefectural Central Hospital for human researches, written informed consent was substituted by the opt-out on the website for obtaining the information in the medical records. We disclosed the details of the study to the patients and publics in the webpage of Shimane Prefectural Central Hospital and informed the patients of their right to refuse enrollment. All study methods were carried out based on the Japanese Ethical Guidelines for Medical and Health Research involving Human Subjects and Declaration of Helsinki.

### Development of CDSS

The electronic medical records with computerized ordering system at Shimane Prefectural Central Hospital is an Integrated Intelligent Management System (IIMS), consisting of electronic medical records, order entry, nursing logs, laboratory and imaging results, prescription data, and hospital claims. We developed the CDSS for glucocorticoid-induced osteoporosis. The physician investigators designed the CDSS and the system engineers constructed the additional codes of the CDSS working on the IIMS. The CDSS obtains the information of prescription data for glucocorticoid for the past 3 months and assess the eligibility of bisphosphonate prescription criteria (Supplementary Table S1) using the master list of glucocorticoid and PSL equivalent doses. If there were no BMD testing for the past year, BMD testing was recommended in the electronic medical record. If a clinician prescribed a glucocorticoid meeting the criteria, an alert would appear in the electronic medical record and these information are stored in the log files (Supplementary Fig. S1). In addition, the CDSS automatically guides BP prescription following the alert. An alert was pop-up displayed each time a clinician prescribed a glucocorticoid meeting the criteria (Supplementary Fig. S2). Physicians could reject the recommendations displayed at their own discretion.

We implemented the CDSS in the IIMS on October 1st, 2018. The CDSS worked in the background until September 30th, 2018 and no alert was displayed, but the IIMS collected the data on the potential alerts. Therefore, physicians could not use the CDSS until September 30th, 2018. After October 1st, 2018, the CDSS alerts were displayed on the IIMS, and physicians were guided by the CDSS and could see the alerts and decide whether to accept guided BP prescription and BMD testing.

### Data collection and outcomes

Patient characteristics were collected on the earliest index day during phase 1 and phase 2 separately. All prescription and laboratory data were collected from the IIMS, as well as data on all hidden and displayed alerts and responses by physicians to the alerts. The data consisted of patient-level and prescription-level data. Patient-level data included age, sex, body mass index (BMI), laboratory data, primary diagnosis for prescription of glucocorticoid, specialty of physician in charge, and the PSL equivalent dose of the prescribed glucocorticoid. We did not collect racial information because the race was not included in the variables in the guideline for glucocorticoid-induced osteoporosis^15^. The primary diagnosis was categorized into rheumatic diseases, hematologic diseases, renal and urogenital diseases, respiratory diseases, intestinal and hepatobiliary diseases, cancer, neurological diseases, endocrine diseases, dermatological diseases, and post organ transplantation (Supplementary Table S2). Physician specialty was divided into general internal medicine (GIM), subspecialty of internal medicine other than GIM, surgery, and others. Prescription-level data included the kinds and doses of glucocorticoid or BP, orders for BMD testing, and alerts for BP prescription and BMD testing.

The outcome measures were the number of alerts and the actual orders for BP prescription and BMD testing. The acceptance rates were calculated by the number of actual orders for BP prescription or BMD testing divided by the number of alerts for BP prescription or BMD testing, respectively. The alerts for BP prescription and BMD testing were hidden alerts that remained in the background and did not appear on the display in phase 1; thus, orders for BP prescription and BMD testing in phase 1 were considered voluntary.

### Statistical analyses

We presented the patient-level variables with medians and interquartile range (IQR) for continuous variables and numbers and percentage for categorical variables stratified by phase 1 and phase 2. We compared patient characteristics between phase 1 and phase 2 using the Chi-squared test for categorical variables and the Wilcoxon rank-sum test for continuous variables. The outcome measures were compared between phase 1 and phase 2 with the Chi-squared test at the patient level and the order level. We used the Fisher’s exact test instead of the Chi-squared test for 2 × 2 contingency table comparison if the expected value in any cell was less than 5. The numbers of alerts for BP prescription or BMD testing were compared between phase 1 and phase 2. The BP prescription rate and the BMD testing rate were calculated as order per alert and compared between phase 1 and phase 2. The numbers of orders for BP and BMD testing monthly were also compared before and after implementing the CDSS.

In phase 2, the patient-level variables were compared between those with and without BP prescription among those who received alerts, using the Chi-squared test for categorical variables and the Wilcoxon rank-sum test for continuous variables. The same analyses were done between those with and without BMD testing.

Adjusted analyses for patient characteristics were not performed, because their characteristics were considered similar between phase 1 and phase 2. All statistical analyses were performed using JMP version 14.2 (SAS Institute, Cary, NC). All reported p-values were two-tailed, and p-values less than 0.05 were considered significant.

### Human subjects protection

This study was approved by the Institutional Review Board at Hyogo College of Medicine (2495) and Shimane Prefectural Central Hospital (R16-064).

## Results

### Patients’ characteristics

**Figure 1:**
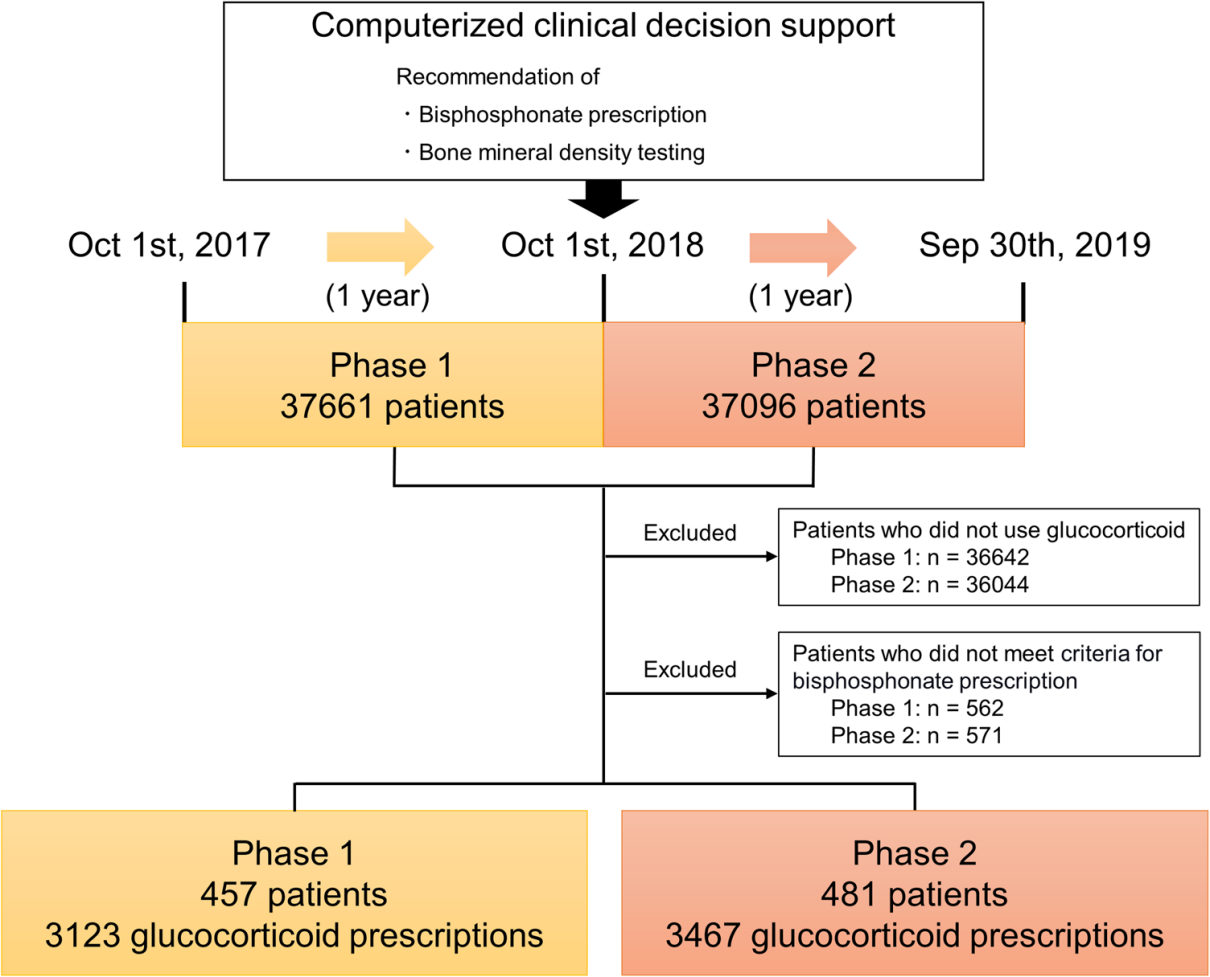
Flowchart of study design and enrolled patients.

During the study period, there were a total of 74,757 outpatients (phase 1, 37,661; phase 2, 37,096), and 938 patients (phase 1, 457 patients; phase 2, 481 patients) met the inclusion criteria and were included (Fig. 1). The patients’ baseline characteristics were similar between phase 1 and phase 2, and the median age was 71 years in both phase 1 and phase 2. The doctors prescribing a glucocorticoid were primarily subspecialists of internal medicine, followed by surgery and GIM (Table 1). The primary disease for prescription of glucocorticoids was rheumatic diseases (27%), followed by hematologic diseases (19%), renal diseases (12%), respiratory diseases (11%), intestinal and hepatobiliary diseases (9%), cancer (8%), neurological diseases (5.5%), and endocrine diseases (5.3%) (Table 1 and Supplementary Table S2). The most frequently prescribed glucocorticoid type was prednisolone, followed by hydroxycorticosterone, betamethasone, dexamethasone, and methylprednisolone. The baseline laboratory data, including renal function and liver function, were also similar between phase 1 and phase 2 (Table 1). The number of glucocorticoid prescriptions was 3123 among the 457 patients in phase 1 and 3467 among the 481 patients in phase 2.

**Table 1:** Patient characteristics.

Variables	Phase 1 (n = 457)	Phase 2 (n = 481)	P value
Age-years, median [IQR]	71 [62–79.5]	71 [64–80]	0.4
Men, n (%)	232 (51)	245 (51)	1.0
Body mass index, median [IQR]	21.7 [19.3–24.5] (n = 290)	21.9 [19.5–24.3] (n = 206)	0.6
**Diseases**			
Rheumatic diseases, n (%)	124 (27)	138 (29)	0.6
Hematologic disease, n (%)	86 (19)	80 (17)	0.4
Renal and urogenital diseases, n (%)	54 (12)	55 (11)	0.9
Respiratory diseases, n (%)	52 (11)	53 (11)	0.9
Intestinal and hepatobiliary diseases, n (%)	42 (9.2)	45 (9.4)	1.0
Cancer, n (%)	36 (7.9)	43 (8.9)	0.6
Neurological disease, n (%)	25 (5.5)	26 (5.4)	1.0
Endocrine diseases, n (%)	24 (5.2)	23 (4.8)	0.8
Dermatological diseases, n (%)	7 (1.5)	12 (2.5)	0.3
Post organ transplantation, n (%)	7 (1.5)	6 (1.2)	0.8
Glucocorticoid type*			
Prednisolone, n (%)	396 (87)	424 (88)	0.5
Prednisolone-mg/day, median [IQR]	7 [5–10]	5 [5–10]	0.2
Hydroxycorticosterone, n (%)	23 (5.0)	23 (4.8)	0.9
Hydroxycorticosterone-mg/day, median [IQR]	20 [10–30]	20 [10–25]	0.6
Betamethasone, n (%)	22 (4.8)	20 (4.2)	0.6
Betamethasone-mg/day, median [IQR]	1.0 [0.5–1.1]	0.9 [0.3–1.8]	0.9
Dexamethasone, n (%)	14 (3.1)	19 (4.0)	0.5
Dexamethasone-mg/day, median [IQR]	12 [4–20]	12 [3–20]	0.5
Methylprednisolone, n (%)	8 (1.8)	5 (1.0)	0.4
Methylprednisolone-mg/day, median [IQR]	2 [2–4]	2 [2–3]	0.5
Prednisolone equivalent dose-mg/day, median [IQR]	7 [5–10]	5 [5–10]	0.3
**Laboratory data**			
Hematocrit-%, median [IQR]	39.4 [35–43] (n = 439)	38.6 [35–42] (n = 457)	0.06
Aspartate aminotransferase-U/l, median [IQR]	21 [17–28] (n = 434)	22 [17–28] (n = 457)	0.9
Alanine aminotransferase-U/l, median [IQR]	17 [12–25] (n = 434)	16 [12–25] (n = 457)	0.3
Lactate dehydrogenase-U/l, median [IQR]	219 [188–261] (n = 410)	217 [185–260] (n = 429)	0.7
Alkaline phosphatase-U/l, median [IQR]	227 [174–293] (n = 360)	218 [168–280] (n = 387)	0.3
Total bilirubin-mg/dL, median [IQR]	0.7 [0.5–0.9] (n = 384)	0.7 [0.5–0.9] (n = 419)	0.8
γ-Glutamyl transpeptidase-U/l, median [IQR]	29 [18–55] (n = 338)	27 [18–48] (n = 378)	0.2
Serum calcium-mg/dL, median [IQR]	9.2 [8.8–9.5] (n = 259)	9.2 [8.9–9.6] (n = 307)	0.2
Blood urea nitrogen-mg/dL, median [IQR]	16 [13–20] (n = 437)	16 [13–20] (n = 457)	1.0
Creatinine-mg/dL, median [IQR]	0.7 [0.6–0.9] (n = 442)	0.75 [0.6–0.9] (n = 461)	0.9
Estimated glomerular filtration rate-mL/min/1.73 m^2^, median [IQR]	69.6 [55–85] (n = 442)	69.6 [56–83] (n = 461)	0.8
**Division**			
General internal medicine, n (%)	42 (9.2)	54 (11)	0.3
Subspecialty of internal medicine, n (%)	345 (75)	347 (72)	0.3
Surgery, n (%)	57 (12)	68 (14)	0.6
Others, n (%)	13 (2.8)	12 (2.5)	0.7
Postgraduate year of physician in charge-years, median [IQR]	23.5 [17–32] (n = 452)	22 [18–31] (n = 474)	0.2

*IQR* interquartile range.

*Patients received multiple glucocorticoid types.

### Effects of the CDSS on BP prescriptions

The prevalence of patients who needed an alert for BP prescription did not change after implementation of the CDSS, from 306 patients (67%) in phase 1 to 304 patients (63%) in phase 2 (P = 0.24). The prevalence of patients who were prescribed BPs was also similar, 48 (16%) in phase 1 and 57 (19%) in phase 2 (P = 0.33) (Table 2).

**Table 2:** Alerts from computerized clinical decision support system.

	Phase 1	Phase 2	P value
**Number of patients who met criteria**	457	481	
Patients who received alert of BP prescription, n (%)	306 (67) [Hidden alert]	304 (63)	0.24
Patients who were ordered BP, n (% per alerted patients)	48 (16) [Order without alert]	57 (19)	0.33
Patients who received alert of BMD testing, n (%)	424 (93) [Hidden alert]	419 (87)	0.004
Patients who were ordered BMD testing, n (% per alerted patients)	18 (4.2) [Order without alert]	101 (24)	< 0.001
**Number of glucocorticoid prescriptions of patients who met criteria**	3123	3467	
Alert of BP prescription, n (%)	1763 (56) [Hidden alert]	1813 (52)	< 0.001
BP prescription, n (% per alert)	97 (5.5) [Order without alert]	113 (6.2)	0.35
Alert of BMD testing, n (%)	2763 (88) [Hidden alert]	2131 (61)	< 0.001
BMD testing, n (% per alert)	14 (0.5) [Order without alert]	112 (5.3)	< 0.001

*BP* bisphosphonates, *BMD* bone mineral density.

**Figure 2:**
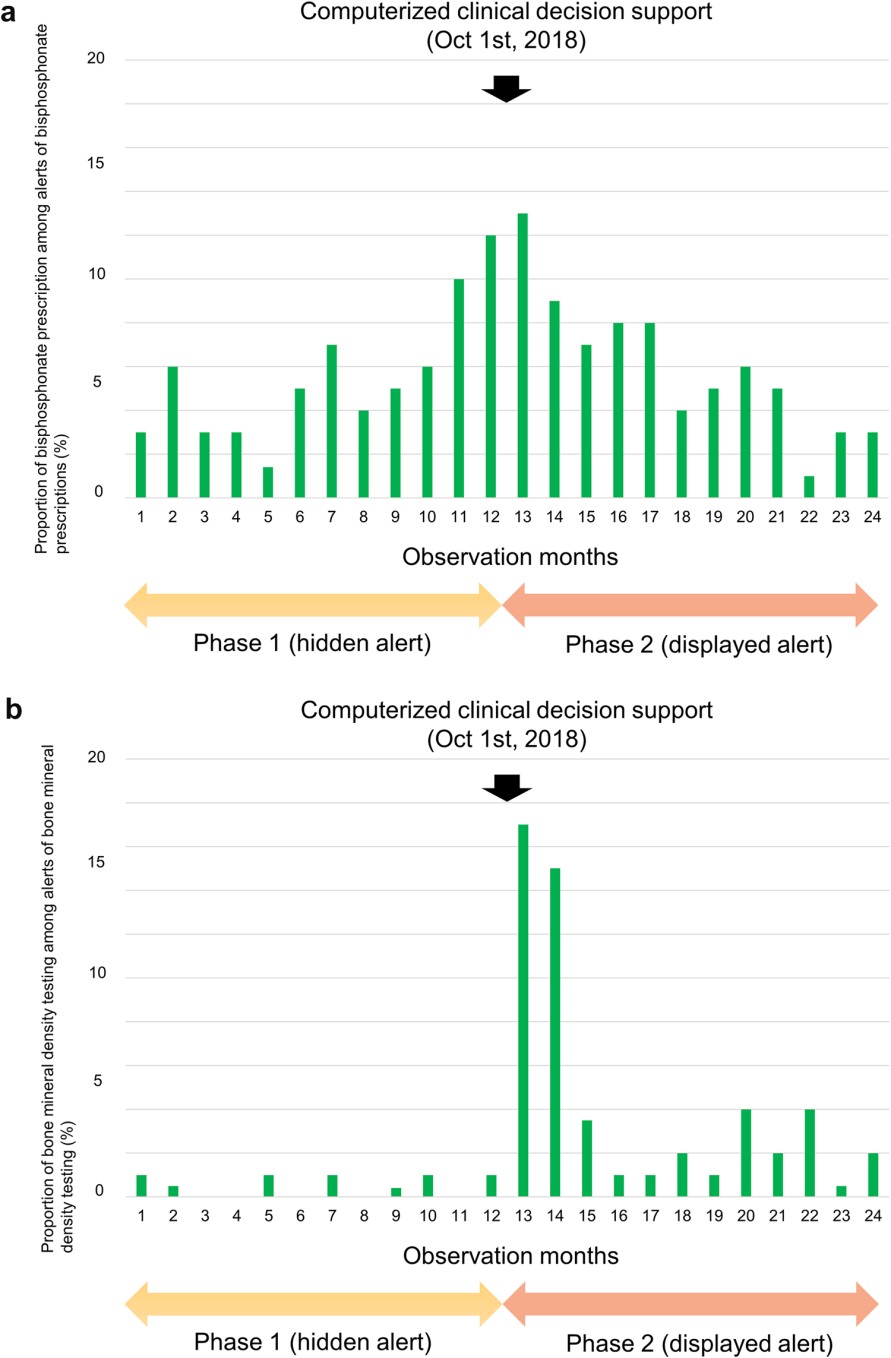
Time trend of prevention of glucocorticoid-induced osteoporosis. (**a**) Bisphosphonate prescription. (**b**) Bone mineral density testing.

The proportion of alerts for BP prescription with glucocorticoid prescription was significantly decreased when the alert was actually displayed in phase 2 (56% vs. 52%, P < 0.001). The acceptance rate of BP prescriptions for patients was similar between phase 1 (5.5%) and phase 2 (6.2%) (P = 0.35) (Table 2). There were no changes in the acceptance rates of BP prescription after the implementation of the CDSS (Fig. 2a).

### Effects of CDSS on BMD testing

The prevalence of patients who needed an alert for BMD testing decreased significantly after implementation of the CDSS, from 424 patients (93%) in phase 1 to 419 patients (87%) in phase 2 (P = 0.004). On the other hand, the prevalence of patients who had BMD testing was significantly increased, from 18 (4.2%) in phase 1 to 101 (24%) in phase 2 (P < 0.001) (Table 2).

The proportion of alerts for BMD testing with glucocorticoid prescription was significantly decreased when the alert was actually displayed in phase 2 (88% vs. 61%, P < 0.001). The acceptance rate of BMD testing was significantly increased from 14 (0.5%) in phase 1 to 112 (5.3%) in phase 2 (P < 0.001) (Table 2). The number of BMD tests increased in the month just after the implementation of the CDSS, and then gradually decreased over time (Fig. 2b).

### Factors related to accepting the alerts from the CDSS

**Figure 3:**
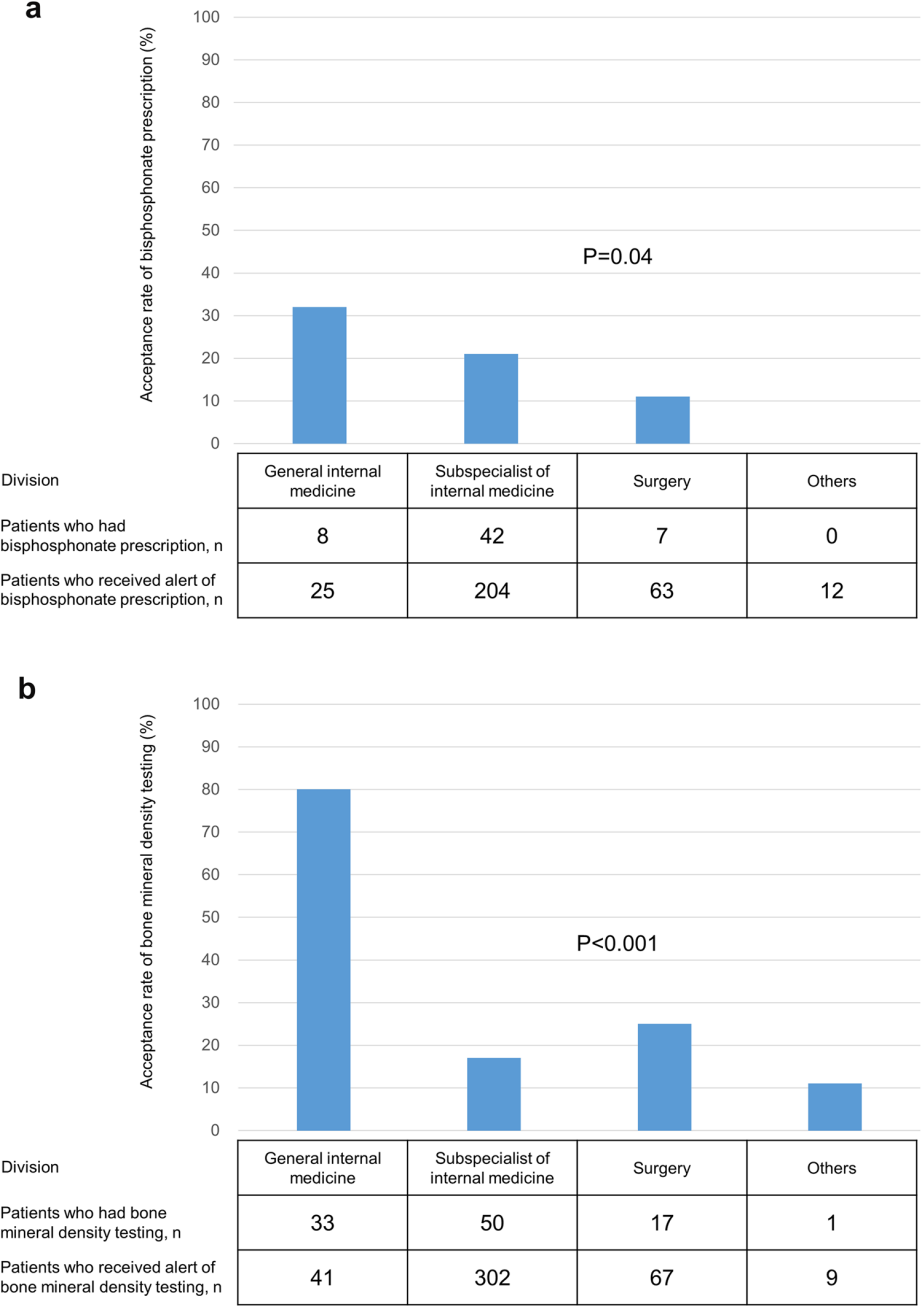
Acceptance rate of alerts for prevention of glucocorticoid-induced osteoporosis stratified by division. (**a**) Bisphosphonate prescription. (**b**) Bone mineral density testing.

The patients' characteristics were generally similar between those who did and did not receive BP prescriptions after alerts in phase 2 (Supplementary Table S3). However, patients with prednisolone were more prevalent among those who received BP prescriptions (P = 0.045). The acceptance rate of a BP prescription alert was highest in the GIM division (32%) and lowest in others (0%), and the difference among divisions was significant (P = 0.03) (Fig. 3a).

The patients’ characteristics were generally similar between those who did and did not have BMD testing after alerts in phase 2 (Supplementary Table S4). However, patients with prednisolone were more prevalent among those who had BMD testing (P = 0.01). In addition, patients who were treated at the GIM division were significantly more likely to have BMD testing (80%) than other divisions (P < 0.001) (Fig. 3b).

The years after graduation of the physicians in charge were not associated with the acceptance rates of BP prescription and BMD testing (Supplementary Tables S3 and Table S4).

## Discussion

We demonstrated that the implementation of a CDSS significantly increased BMD testing in patients with a higher risk of glucocorticoid-induced osteoporosis based on a CPG, but did not increase BP prescriptions. The ordering of BMD testing increased just after implementation of the CDSS, and it then gradually decreased over time with the CDSS. Among patients who had alerts for BMD testing, the acceptance rate was 24%, and it was higher among patients on prednisolone than other glucocorticoids or patients treated by physicians in GIM than physicians in other divisions.

One previous study also showed that a CDSS significantly improved the order rates of BMD testing from 5.9% before to 9.8% after implementing a CDSS for women with primary osteoporosis who did not have baseline BMD testing^16^. That study was conducted in a primary care setting, and preventive care for osteoporosis was the primary interest of the provider. Although the setting of the present study was a tertiary care hospital with a mixed case load, and most providers were subspecialists, the improvement of BMD testing from 4 to 24% after implementation of the CDSS was much larger than in the previous study. This larger improvement was especially driven by the higher acceptance rate among physicians in GIM. A gap in acceptance of alerts from a CDSS between GIM and subspecialty physicians was reported in the US, which demonstrated that physicians in a medical subspecialty were 17% less likely to accept alerts for drug interactions compared to family or internal medicine physicians^17^. These observations imply that physicians in the subspecialties of internal medicine take responsibility for their primary diseases, rather than preventive care of osteoporosis, and physicians in GIM or family medicine try to cover problems outside their primary interest.

However, there was no significant improvement in the BP prescription rate. A previous study had shown that a CDSS improved the BP prescription rate for primary osteoporosis in patients who had a low T-score (less than − 2.5) and a history of hip or vertebral fracture in the primary care setting^18^. This result could suggest that physicians might be likely to accept the recommendation by a CDSS when the target patients have a clear indication for BP. Another possible reason could be that physicians hesitate to prescribe because they are concerned about the adverse drug events of anti-osteoporosis drugs. The well-known and critical adverse events of anti-osteoporosis drugs was bisphosphonate-associated osteonecrosis of the jaw or atypical femur fractures^19,20^. Concerns about such adverse drug events due to BP or the need for dental consultation might be barriers to prescribing BP based on CDSS-generated alerts alone.

CDSSs have been reported to improve process measures such as BP prescription rates in primary osteoporosis^21^, but no studies have reported whether a CDSS improved patient outcomes such as fractures. It would take thousands of patients and several years to demonstrate the effectiveness of a CDSS to reduce incidences of rare events. Therefore, it was inevitable that the effect of a CDSS on glucocorticoid-induced osteoporosis would have to be evaluated with process measures, such as BMD testing and BP prescription, in the present study. On the other hand, BP prescription has been shown to reduce vertebral or hip fractures in patients with glucocorticoid-induced osteoporosis in major clinical trials^22^. In other settings, CDSSs were shown to be effective to prevent venous thromboembolism with appropriate prescription of anticoagulants^7^. Thus, CDSSs may decrease fractures with evidence-based preventive measures when appropriately implemented and accepted in clinical settings. Another report showed that BMD screening combined with BP prescription was highly cost-effective in primary osteoporosis, and such combination of BMD screening and BP prescription may be effective in glucocorticoid-induced osteoporosis^23^. Further studies to confirm the effectiveness of CDSSs for glucocorticoid-induced osteoporosis should be considered.

Beyond glucocorticoid-induced osteoporosis, CDSSs have the potential to improve the application rate of CPGs^24^. CDSSs have been reported to improve the adherence rate to CPGs in asthma practice^9^ and the appropriate prescription rate for renal impairment^25^. Such documentation, test ordering, and adjustment of pre-existing drugs with CDSSs could be relatively easily accepted by healthcare providers. However, starting a new drug according to a CDSS is a high hurdle, because physicians tend to continue current practice if there are no apparent needs to change^25^. The additional target of CDSSs could be such new prescription orders based on CPGs.

This study had several limitations. First, this was an observational study to compare practice before and after implementation of a CDSS. The effects of factors other than the CDSS could not be excluded. Especially, factors in patients should not be ignored. Even if the CDSS recommended and physicians accepted the alerts, patients could refuse the recommendation. As a result, the acceptance rate could be lowered by the patients’ refusal. However, this study was conducted at a single center for a relatively short period of 2 years, and the clinical situation did not change over time. The attitude of patients was also less likely to change during the study period. Indeed, the patients’ characteristics were similar between phases. Second, this study did not evaluate clinical outcomes, such as the incidence of hip or vertebral fractures, when using the CDSS. However, the relationship between low BMD and subsequent fractures has been well documented^26^. Therefore, the usage of BMD testing or BP prescription as outcomes in this study should be acceptable. Third, practice outside the study hospital might have been missed, including medications or examinations at other hospitals. However, the study hospital was the local core hospital, and missing such information was less likely. Fourth, we did not incorporate the data on the history of previous fractures in the CDSS because the history of previous fractures was not systematically documented in the medical record in the outpatient setting. The history of previous fractures is an apparent indication for osteoporosis prevention and thus the benefit of CDSS might not be large. The history of previous fracture is also used to calculate Fracture Risk Assessment Tool (FRAX) and FRAX is easily used to estimate the 10-year risk of fracture^27^. Although we did not consider the FRAX in this study, we should investigate the efficacy of CDSS incorporating the history of previous fracture or FRAX in the future studies. Finally, this study was conducted at a single center, which limits its generalizability. In addition, the implemented CDSS was developed in this study. Although the decision pathways of the CDSS were simple enough that any electronic medical records or ordering systems could deploy it, the findings should be confirmed in other settings worldwide.

## Conclusion

A CDSS could improve glucocorticoid-induced osteoporosis practice based on the CPG. The performance rate of guided practice was still less than perfect; thus, further investigation should be conducted to improve the performance rate of guided practice, as well as the patients’ outcomes.

## Supplementary Information

Supplementary Information.

## Data Availability

The datasets used and/or analyzed during the current study are available from the corresponding author on reasonable request

## References

[1] Shekelle PG (2018). Clinical practice guidelines: What's next?. JAMA.

[2] Jin YH (2021). Determinants of successful guideline implementation: A national cross-sectional survey. BMC Med. Inform. Decis. Mak..

[3] Grol R (1998). Attributes of clinical guidelines that influence use of guidelines in general practice: Observational study. BMJ.

[4] Boyd CM (2005). Clinical practice guidelines and quality of care for older patients with multiple comorbid diseases: Implications for pay for performance. JAMA.

[5] Hunt DL, Haynes RB, Hanna SE, Smith K (1998). Effects of computer-based clinical decision support systems on physician performance and patient outcomes: A systematic review. JAMA.

[6] Kucher N (2005). Electronic alerts to prevent venous thromboembolism among hospitalized patients. N. Engl. J. Med..

[7] Durieux P, Nizard R, Ravaud P, Mounier N, Lepage E (2000). A clinical decision support system for prevention of venous thromboembolism: Effect on physician behavior. JAMA.

[8] Kwok R, Dinh M, Dinh D, Chu M (2009). Improving adherence to asthma clinical guidelines and discharge documentation from emergency departments: Implementation of a dynamic and integrated electronic decision support system. Emerg. Med. Aust..

[9] Gudmundsson HT, Hansen KE, Halldorsson BV, Ludviksson BR, Gudbjornsson B (2019). Clinical decision support system for the management of osteoporosis compared to NOGG guidelines and an osteology specialist: A validation pilot study. BMC Med. Inform. Decis. Mak..

[10] Buckley L (2017). 2017 American College of Rheumatology Guideline for the prevention and treatment of glucocorticoid-induced osteoporosis. Arthritis Rheumatol..

[11] Majumdar SR (2012). Population-based trends in osteoporosis management after new initiations of long-term systemic glucocorticoids (1998–2008). J. Clin. Endocrinol. Metab..

[12] Kastner M, Straus SE (2008). Clinical decision support tools for osteoporosis disease management: A systematic review of randomized controlled trials. J. Gen. Intern. Med..

[13] Klop C (2014). Increase in prophylaxis of glucocorticoid-induced osteoporosis by pharmacist feedback: A randomised controlled trial. Osteoporos. Int..

[14] Solomon DH, Katz JN, La Tourette AM, Coblyn JS (2004). Multifaceted intervention to improve rheumatologists' management of glucocorticoid-induced osteoporosis: A randomized controlled trial. Arthritis Rheum..

[15] Suzuki Y (2014). Guidelines on the management and treatment of glucocorticoid-induced osteoporosis of the Japanese Society for Bone and Mineral Research: 2014 update. J. Bone Miner. Metab..

[16] DeJesus RS (2012). Use of a clinical decision support system to increase osteoporosis screening. J. Eval. Clin. Pract..

[17] Isaac T (2009). Overrides of medication alerts in ambulatory care. Arch Intern. Med..

[18] Goldshtein I (2020). Development and efficacy of a computerized decision support system for osteoporosis management in the community. Arch Osteoporos..

[19] Mavrokokki T, Cheng A, Stein B, Goss A (2007). Nature and frequency of bisphosphonate-associated osteonecrosis of the jaws in Australia. J. Oral. Maxillofac. Surg..

[20] Black DM, Abrahamsen B, Bouxsein ML, Einhorn T, Napoli N (2019). Atypical femur fractures: Review of epidemiology, relationship to bisphosphonates, prevention, and clinical management. Endocr. Rev..

[21] Homik J (2000). Bisphosphonates for steroid induced osteoporosis. Cochrane Database Syst. Rev..

[22] Schousboe JT, Ensrud KE, Nyman JA, Melton LJ, Kane RL (2005). Universal bone densitometry screening combined with alendronate therapy for those diagnosed with osteoporosis is highly cost-effective for elderly women. J. Am. Geriatr. Soc..

[23] Sutton RT (2020). An overview of clinical decision support systems: Benefits, risks, and strategies for success. NPJ Digit. Med..

[24] Wada R (2020). Clinical decision support system with renal dose adjustment did not improve subsequent renal and hepatic function among inpatients: The Japan Adverse Drug Event Study. Appl. Clin. Inform..

[25] O'Connor, P. J., Sperl-Hillen, J. M., Johnson, P. E., Rush, W. A. & Biltz, G. in *Advances in Patient Safety: From Research to Implementation (Volume 2: Concepts and Methodology)* (eds K. Henriksen, J. B. Battles, E. S. Marks, & D. I. Lewin) (Agency for Healthcare Research and Quality (US), 2005). 21249825

[26] Ferrari S (2019). Relationship between bone mineral density T-score and Nonvertebral fracture risk over 10 years of denosumab treatment. J. Bone Miner. Res..

[27] Kanis JA, Johnell O, Oden A, Johansson H, McCloskey E (2008). FRAX and the assessment of fracture probability in men and women from the UK. Osteoporos Int..

