# Supplementary material for: Effectiveness of a computerized clinical decision support system for prevention of glucocorticoid-induced osteoporosis
Source: Sci Rep. 2022 Sep 2;12:14967. doi: 10.1038/s41598-022-19079-7 (PMC9440130; doi:10.1038/s41598-022-19079-7)
Supplement: Supplementary file 1 — Supplementary Information. [file 41598_2022_19079_MOESM1_ESM.pdf]

Supplementary Tables and Figures

Supplementary Figure S1. The structure of the clinical decision support system

Supplementary Figure S2. Alert displayed on medical record

Supplementary Table S1. Criteria for bisphosphonate prescription

Supplementary Table S2. Diseases for steroid prescriptions

Supplementary Table S3. Patients with and without bisphosphonates prescription who received alerts in phase 2

Supplementary Table S4. Patients with and without bone mineral density testing who received alerts in phase 2

Supplementary Figure S1. The structure of the clinical decision support system

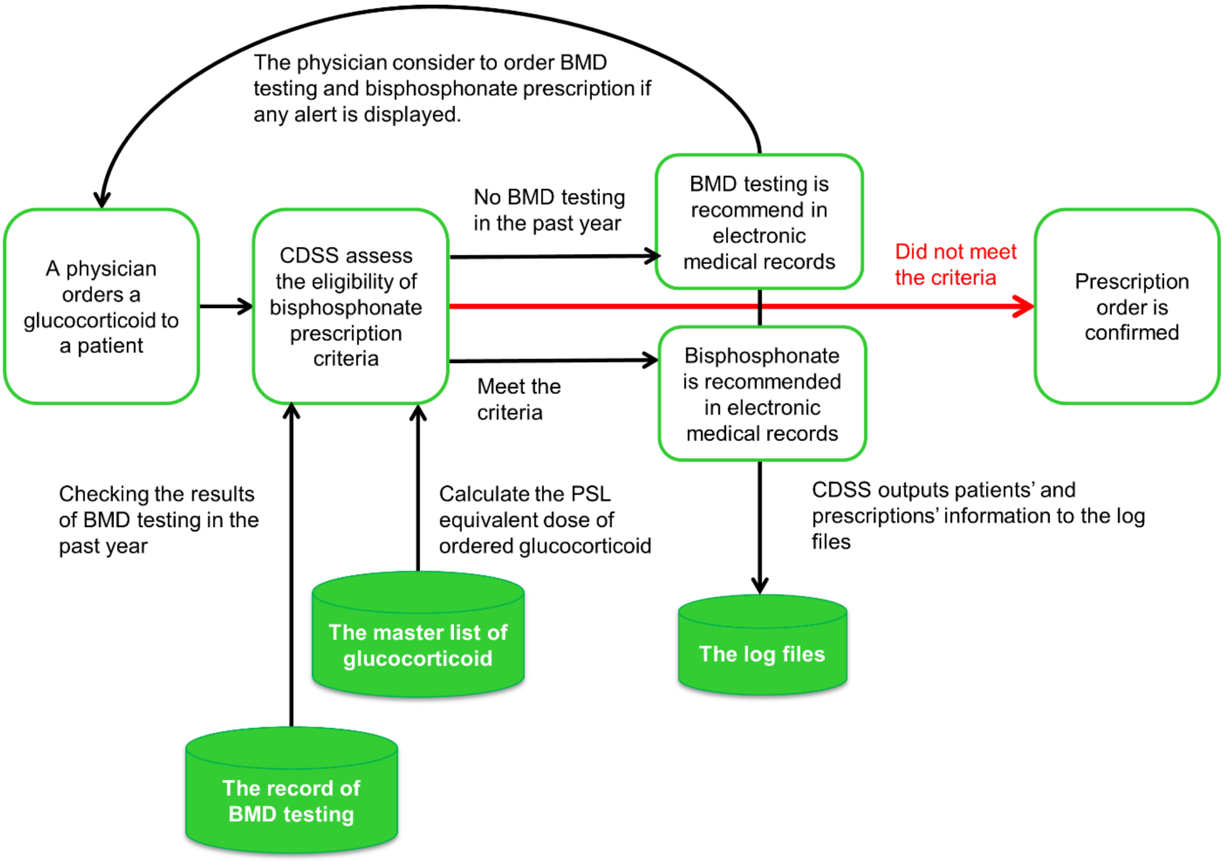

CDSS, clinical decision support system; BMD, bone mineral density; PSL, prednisolone

Supplementary Figure S2. Alert displayed on medical record

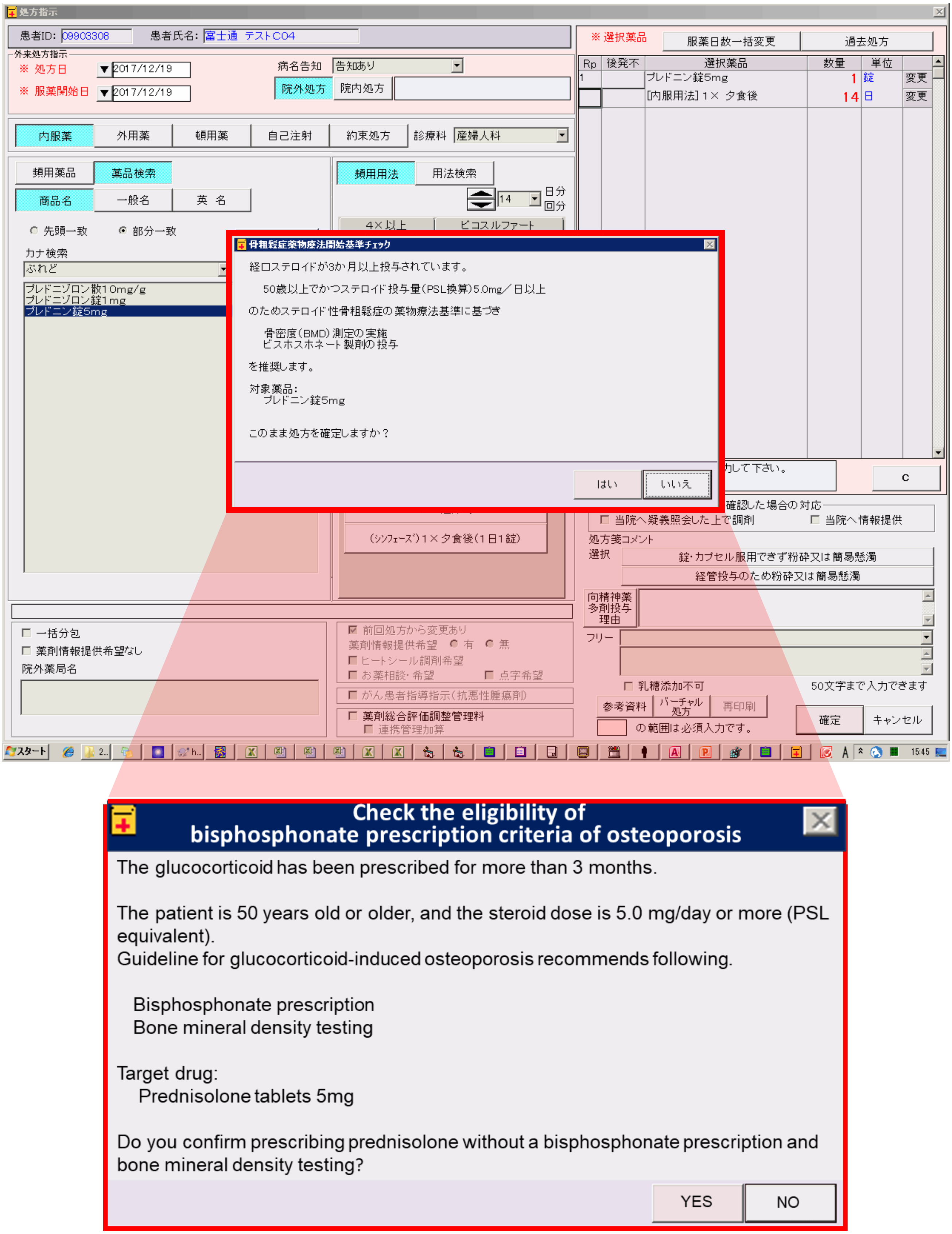

Supplementary Table S1. Criteria for bisphosphonate prescription

**Patients on oral steroids for at least 3 months with any of the following criteria**	
1	Steroid dose (PSL equivalent) of 7.5 mg/day or more
2	65 years or older
3	50 years or older and steroid dose (PSL equivalent) 5 mg/day or higher
4	Bone mineral density less than YAM 70% [a]
5	Bone mineral density YAM 70-80% and steroid dose 5.0 mg/day or more [a]
6	Bone mineral density YAM 70-80% and over 50 years [a]

PSL, prednisolone; YAM, young adult mean.

[a] Bone mineral density was judged by the most recently tested data in the past one year.

Supplementary Table S2. Diseases for steroid prescriptions

Diseases	Phase 1 (n = 457)	Phase 2 (n = 481)
Rheumatic diseases, n (%)	124 (27)	138 (29)
Polymyalgia rheumatica	30 (6.6)	30 (6.2)
Rheumatoid arthritis	22 (4.8)	24 (5.0)
Anti-neutrophil cytoplasmic antibody-associated vasculitis	16 (3.5)	25 (5.2)
Systemic lupus erythematosus	14 (3.1)	15 (3.1)
Polymyositis/ Dermatomyositis	8 (1.8)	8 (1.7)
Sarcoidosis	6 (1.3)	7 (1.5)
IgG4-related disease	6 (1.3)	6 (1.2)
IgA vasculitis	4 (0.9)	3 (0.6)
Large vasculitis	3 (0.7)	4 (0.8)
Other vasculitis	3 (0.7)	3 (0.6)
Behçet's disease	2 (0.4)	3 (0.6)
Adult onset still disease	2 (0.4)	2 (0.4)
Sjogren's syndrome	2 (0.4)	2 (0.4)
Mixed connective tissue disease	2 (0.4)	1 (0.2)
Remitting seronegative symmetrical synovitis with pitting oedema syndrome	1 (0.2)	2 (0.4)
Relapsing polychondritis	1 (0.2)	1 (0.2)
Gout/ Pseudo Gout	1 (0.2)	1 (0.2)
Periodic fever with aphthous stomatitis, pharyngitis, and adenitis	1 (0.2)	1 (0.2)
Hematologic disease, n (%)	86 (19)	80 (17)
Multiple myeloma	23 (5.0)	23 (4.8)
Idiopathic thrombocytopenic purpura	17 (3.7)	11 (2.3)
Autoimmune hemolytic anemia	9 (2.0)	11 (2.3)
Malignant lymphoma	9 (2.0)	6 (1.2)
Leukemia	8 (1.8)	9 (1.9)
Hypereosinophilic syndrome	8 (1.8)	8 (1.8)
Myelodysplastic syndrome	7 (1.5)	8 (1.7)
Myelofibrosis	2 (0.4)	2 (0.4)
Aplastic anemia	1 (0.2)	1 (0.2)
Polyneuropathy, organomegaly, endocrinopathy, monoclonal protein and skin changes syndrome	1 (0.2)	1 (0.2)
Thrombotic thrombocytopenic purpura	1 (0.2)	0 (0.0)
Renal and urogenital diseases, n (%)	54 (12)	55 (11)
Nephrotic syndrome	28 (6.1)	27 (5.6)
IgA nephropathy	17 (3.7)	17 (3.5)

Retroperitoneal fibrosis	4 (0.9)	3 (0.6)
Glomerulonephritis	3 (0.7)	6 (1.2)
Interstitial nephritis	2 (0.4)	2 (0.4)
Respiratory diseases, n (%)	52 (11)	53 (11)
Interstitial pneumonia	22 (4.8)	25 (5.2)
Organized pneumonia	12 (2.6)	9 (1.9)
Drug-induced interstitial lung disease	5 (1.1)	6 (1.2)
Hypersensitivity pneumonitis	3 (0.7)	3 (0.6)
Radiation pneumonitis	3 (0.7)	1 (0.2)
Eosinophilic pneumonia	2 (0.4)	3 (0.6)
Lymphangioleiomyomatosis	2 (0.4)	2 (0.4)
Eosinophil sinusitis	1 (0.1)	2 (0.4)
Bronchial asthma	1 (0.2)	1 (0.2)
Sclerotic mediastinitis	1 (0.2)	1 (0.2)
Intestinal and hepatobiliary diseases, n (%)	42 (9.2)	45 (9.4)
Autoimmune hepatitis	26 (5.7)	28 (5.8)
Autoimmune pancreatitis	6 (1.3)	5 (1.0)
Inflammatory bowel disease	5 (1.1)	10 (2.1)
Eosinophilic gastroenteritis	2 (0.4)	1 (0.2)
Cronkhite-canada syndomer	1 (0.2)	1 (0.2)
Drug induced gastroenteritis	1 (0.2)	0 (0.0)
Encapsulating peritoneal sclerosis	1 (0.2)	0 (0.0)
Cancer, n (%)	36 (7.9)	43 (8.9)
Lung cancer	13 (2.8)	19 (4.0)
Prostate cancer	12 (2.6)	17 (3.5)
Gastric cancer	3 (0.7)	0 (0.0)
Breast cancer	2 (0.4)	0 (0.0)
Pancreatic cancer	2 (0.4)	1 (0.2)
Colon cancer	1 (0.2)	3 (0.6)
Brain cancer	1 (0.2)	1 (0.2)
Malignant pleural mesothelioma	1 (0.2)	1 (0.2)
Bladder cancer	1 (0.2)	0 (0.0)
Duodenal papilla carcinoma	0 (0.0)	1 (0.2)
Neurological disease, n (%)	25 (5.5)	26 (5.4)
Myasthenia gravis	11 (2.4)	13 (2.7)
Chronic inflammatory demyelinating polyneuropathy	4 (0.9)	2 (0.4)
Optic neuritis	2 (0.4)	2 (0.4)
Encephalitis	2 (0.4)	2 (0.4)
Neuromyelitis optica	2 (0.4)	2 (0.4)
Peripheral nerve palsy	2 (0.4)	2 (0.4)
Multiple sclerosis	1 (0.2)	2 (0.4)
Guillain barre syndrome	1 (0.2)	0 (0.0)

Hypertrophic pachymeningitis	0 (0.0)	1 (0.2)
Endocrine diseases, n (%)	24 (5.2)	23 (4.8)
Adrenal insufficiency	23 (5.0)	22 (4.6)
Hyperthyroidism	1 (0.2)	1 (0.2)
Dermatological diseases, n (%)	7 (1.5)	12 (2.5)
Pemphigoid	5 (1.1)	10 (2.1)
Pemphigus	1 (0.2)	1 (0.2)
Other dermatosis disease	1 (0.2)	1 (0.2)
Post organ transplantation, n (%)	7 (1.5)	6 (1.2)

Supplementary Table S3. Patients with and without bisphosphonates prescription who received alerts in phase 2

Variables	With BP prescription (n = 57)	Without BP prescription (n = 247)	P value
Age-years, median [IQR]	71 [62.5-80.5]	71 [66-81]	0.5
Men, n (%)	26 (46)	152 (62)	0.03
Body mass index, median [IQR]	22 [18-24] (n = 20)	21 [19-24] (n = 119)	0.8
Diseases			
Rheumatic disease, n (%)	12 (21)	47 (19)	0.7
Hematologic disease, n (%)	13 (23)	41 (17)	0.3
Renal and urogenital diseases, n (%)	4 (7.0)	34 (14)	0.2
Respiratory diseases, n (%)	6 (11)	35 (14)	0.5
Intestinal and hepatobiliary diseases, n (%)	10 (18)	9 (3.6)	<0.001
Cancer, n (%)	6 (11)	37 (15)	0.4
Neurological disease, n (%)	4 (7.0)	11 (4.5)	0.5
Endocrine diseases, n (%)	0 (0.0)	21 (8.5)	0.02
Dermatological diseases, n (%)	1 (1.8)	8 (3.2)	1.0
Post organ transplantation, n (%)	1 (1.8)	4 (1.6)	1.0
Glucocorticoid type [a]			
Prednisolone, n (%)	53 (93)	202 (82)	0.045
Prednisolone dose-mg/day, median [IQR]	5 [4.5-10]	5 [4.8-10]	0.9
Hydroxycorticosterone, n (%)	1 (1.8)	20 (8.1)	0.1
Hydroxycorticosterone-mg/day, median [IQR]	10 [10-10]	20 [11.3-28.8]	0.2
Betamethasone, n (%)	3 (5.3)	15 (6.1)	1.0
Betamethasone-mg/day, median [IQR]	0.5 [0.25-1]	0.8 [0.25-2]	0.6
Dexamethasone, n (%)	0 (0.0)	16 (6.5)	0.049
Dexamethasone-mg/day, median [IQR]	0	12 [8-20]	-
Methylprednisolone, n (%)	1 (1.8)	3 (1.2)	0.6
Methylprednisolone-mg/day, median [IQR]	2 [2-2]	2 [2-4]	0.6
Prednisolone equivalent dose-mg/day, median [IQR]	5 [4-10]	5 [5-10]	0.5
Laboratory data			
Hematocrit-%, median [IQR]	38 [33.5-41]	38 [34-42]	0.9

	(n = 55)	(n = 227)	
Aspartate aminotransferase-U/l, median [IQR]	23 [17-28] (n = 56)	21 [17-28] (n = 227)	0.4
Alanine aminotransferase-U/l, median [IQR]	18 [13-29] (n = 56)	15 [11-23] (n = 227)	0.053
Lactate dehydrogenase-U/l, median [IQR]	221 [188-272] (n = 52)	212 [182-256] (n = 213)	0.2
Alkaline phosphatase-U/l, median [IQR]	213 [165-291] (n = 51)	229 [181-303] (n = 189)	0.2
Total bilirubin-mg/dL, median [IQR]	0.7 [0.5-1] (n = 51)	0.7 [0.5-0.9] (n = 207)	0.97
γ-Glutamyl transpeptidase-U/l, median [IQR]	27.5 [19-61] (n = 48)	28 [17-52] (n = 180)	0.5
Serum calcium-mg/dL, median [IQR]	9.3 [9.0-9.6] (n = 47)	9.2 [8.9-9.6] (n = 157)	0.2
Blood urea nitrogen-mg/dL, median [IQR]	17 [14-22] (n = 56)	16 [13-21] (n = 230)	0.5
Creatinine-mg/dL, median [IQR]	0.76 [0.6-1] (n = 56)	0.79 [0.6-1] (n = 231)	0.5
Estimated glomerular filtration rate-mL/min/1.73m^2, median [IQR]	66 [52-84] (n = 56)	67 [53-83] (n = 231)	0.9
Division			
General internal medicine, n (%)	8 (14)	17 (7)	0.1
Subspeciality of internal medicine, n (%)	42 (74)	162 (65)	0.2
Surgery, n (%)	7 (12)	56 (23)	0.1
Others, n (%)	0 (0.0)	12 (5)	0.1
Postgraduate year of physician in charge-years, median [IQR]	28 [18-34] (n = 56)	23 [18-32] (n = 242)	0.3

IQR, interquartile range; BP, bisphosphonates.

[a] Patients received multiple glucocorticoid types.

Supplementary Table S4. Patients with and without bone mineral density testing who received alerts in phase 2

Variables	With BMD testing (n = 101)	Without BMD testing (n = 318)	P value
Age-years, median [IQR]	71 [67-80]	72 [66-81]	0.9
Men-n (%)	44 (44)	173 (54)	0.06
Body mass index, median [IQR]	22.9 [20.2-25.9] (n = 30)	21.6 [19.6-24.0] (n = 148)	0.1
Diseases			
Rheumatic disease, n (%)	27 (27)	91 (29)	0.7
Hematologic disease, n (%)	12 (12)	66 (21)	0.046
Renal and urogenital diseases, n (%)	8 (7.9)	27 (8.5)	0.9
Respiratory diseases, n (%)	12 (12)	36 (11)	0.9
Intestinal and hepatobiliary diseases, n (%)	19 (19)	16 (5.0)	<0.001
Cancer, n (%)	7 (6.9)	36 (11)	0.2
Neurological disease, n (%)	11 (11)	12 (3.8)	0.01
Endocrine diseases, n (%)	3 (3.0)	20 (6.3)	0.2
Dermatological diseases, n (%)	1 (1.0)	10 (3.1)	0.5
Post organ transplantation, n (%)	1 (1.0)	4 (1.3)	1.0
Glucocorticoid type [a]			
Prednisolone, n (%)	95 (94)	268 (84)	0.01
Prednisolone-mg/day, median [IQR]	5 [5-10]	5 [4-10]	0.26
Hydroxycorticosterone, n (%)	4 (4.0)	19 (6.0)	0.6
Hydroxycorticosterone-mg/day, median [IQR]	15 [10-20]	20 [10-30]	0.4
Betamethasone, n (%)	2 (2.0)	18 (5.7)	0.2
Betamethasone-mg/day, median [IQR]	1.3 [0.5-2]	0.9 [0.25-1.4]	0.6
Dexamethasone, n (%)	2 (2.0)	17 (5.4)	0.27
Dexamethasone-mg/day, median [IQR]	20 [20-20]	8 [2.5-16]	0.08
Methylprednisolone, n (%)	1 (1.0)	3 (0.9)	1.0
Methylprednisolone-mg/day, median [IQR]	2 [2-2]	2 [2-4]	0.6
Prednisolone equivalent dose-mg/day, median [IQR]	5 [5-10]	5 [4-10]	0.17
Laboratory data			
Hematocrit-%, median [IQR]	40 [36-43.6] (n = 96)	38 [34-42] (n = 301)	0.005

Aspartate aminotransferase-U/l, median [IQR]	22 [16-25] (n = 96)	21 [17-28.5] (n = 301)	0.4
Alanine aminotransferase-U/l, median [IQR]	16.5 [12-24] (n = 96)	16 [11-25] (n = 301)	0.9
Lactate dehydrogenase-U/l, median [IQR]	211 [173-256] (n = 91)	219 [188-261] (n = 287)	0.1
Alkaline phosphatase-U/l, median [IQR]	204 [160-266] (n = 78)	220 [169-295] (n = 258)	0.1
Total bilirubin-mg/dL, median [IQR]	0.7 [0.5-0.9] (n = 87)	0.7 [0.5-0.9] (n = 281)	0.7
γ-Glutamyl transpeptidase-U/l, median [IQR]	22 [17-29] (n = 83)	27 [18-52] (n = 252)	0.1
Serum calcium-mg/dL, median [IQR]	9.3 [9.0-9.6] (n = 62)	9.2 [8.8-9.5] (n = 199)	0.1
Blood urea nitrogen-mg/dL, median [IQR]	16 [13-20] (n = 95)	16 [13-20] (n = 302)	0.6
Creatinine-mg/dL, median [IQR]	0.79 [0.6-0.9] (n = 96)	0.76 [0.6-0.9] (n = 305)	0.7
Estimated glomerular filtration rate-mL/min/1.73m^2, median [IQR]	67 [55-78] (n = 96)	70 [57-82] (n = 305)	0.2
Division			
General internal medicine, n (%)	33 (33)	8 (2.5)	<0.001
Subspecialty of internal medicine, n (%)	50 (50)	252 (79)	<0.001
Surgery, n (%)	17 (17)	50 (16)	0.8
Others, n (%)	1 (1.0)	8 (2.5)	0.7
Postgraduate year of physician in charge-years, median [IQR]	22 [16-28.5] (n = 101)	21 [18-33] (n = 311)	0.3

IQR, interquartile range; BMD, bone mineral density.

[a] Patients received multiple glucocorticoid types.
